# Vitamin D Deficiency During Development Permanently Alters Liver Cell Composition and Function

**DOI:** 10.3389/fendo.2022.860286

**Published:** 2022-05-12

**Authors:** Kassidy Lundy, John F. Greally, Grace Essilfie-Bondzie, Josephine B. Olivier, Reanna Doña-Termine, John M. Greally, Masako Suzuki

**Affiliations:** ^1^Department of Genetics, Albert Einstein College of Medicine, Bronx, NY, United States; ^2^Retired, Galway, Ireland

**Keywords:** vitamin D deficiency, prenatal environment, liver, transcriptional alterations, DOHaD (developmental origins of health and disease)

## Abstract

Vitamin D, a fat-soluble vitamin, plays a critical role in calcium homeostasis, the immune system, and normal development. Many epidemiological cohort studies globally have found high prevalence rates of vitamin D deficiency and insufficiency, recognized as an important health issue that needs to be solved. In particular, reproductive age and pregnant women low in vitamin D status may confer risks of diseases like obesity on their offspring. While observational studies have suggested associations between prenatal vitamin D deficiency and metabolic phenotypes in offspring, not yet determined is whether prenatal vitamin D deficiency permanently alters the development of the liver, a major metabolic organ. We tested the histopathology and the transcriptomic profiles of livers from male C57BL/6J mice exposed to prenatal vitamin D deficiency through a maternal dietary intervention model. We found that prenatal vitamin D deficiency increases the prevalence of histopathological changes in the liver, and alters its gene expression profile. Cell subtype proportion analysis showed that the liver of prenatal vitamin D deficiency alters non-parenchymal cells of the liver, specifically macrophages, a subset of endothelial cells, and dendritic cells. Our results indicate the long-term memory of prenatal vitamin D deficiency exposure in the adult liver, a potential contributor to offspring health risks.

## Introduction

Vitamin D is a crucial micronutrient that plays many physiologic functions in mammals. Humans create vitamin D from the action of sunlight on the skin, with the consequence that limiting sun exposure, covering skin with clothes, or darker skin pigmentation reduces vitamin D production and may cause its deficiency. The serum 25-hydroxyvitamin D concentration is the marker of vitamin D nutritional adequacy. Serum 25-hydroxyvitamin D concentrations in the range of 50-125 nmol/L indicate adequate vitamin D intake, 30-49 nmol/L is inadequate, and less than 30 nmol/L is classified as deficient, while greater than 125 nmol/L represents excess (1, 2). Many countries provide recommended daily vitamin D intake values and fortified foods such as milk, margarine, flours, cereals, and juices to reduce the prevalence rate of vitamin D deficiency (VDD), but VDD remains highly prevalent worldwide (3–6). In particular, the prevalence of VDD in reproductive-age women and pregnant women is recognized to be an urgent issue that needs to be addressed in low- and mid-income countries, and in high-income countries within the specific race and ethnicity groups (6–8).

While a major role of vitamin D is in regulating calcium homeostasis [reviewed in (9)], it has broader physiological effects. The bioactive metabolite of vitamin D, 1,25(OH)2D3 (calcitriol), is a steroid hormone, influencing transcriptional regulation through interaction with the high-affinity nuclear vitamin D receptor (VDR), a member of the nuclear receptor superfamily of ligand-activated transcription factors. The VDR pathway regulates the transcription of many genes, including genes with essential roles in fetal development and ensures the fetal supply of calcium for bone development (10–13). Intriguingly, epidemiological studies exploring the effects of maternal vitamin D deficiency revealed that offspring exposed to vitamin D deficiency in the uterus increased not only the risk of infantile rickets (4) but also the risk of childhood obesity (14–17) later in adulthood, suggesting long-term memories of the deficiency while developing *in utero*.

A mouse dietary intervention study revealed that mice born to mothers fed a vitamin D deficient diet had rapid weight gain after weaning and were more susceptible to high-fat diet-induced adipocyte hypertrophy than those born to mothers fed a vitamin D sufficient diet (18). In addition, a study of mice exposed to prenatal VDD found that prenatal VDD alters the DNA methylation status of the liver and sperm of two generations of offspring (19). These findings suggest that prenatal vitamin D deficiency predisposes offspring to long-term metabolic phenotypic alterations. However, little is known if prenatal vitamin D deficiency permanently alters the physiology of the liver, a major metabolic organ. To fill this knowledge gap, we assessed the effects of prenatal vitamin D deficiency on liver physiology and gene expression at the adult stage.

## Materials And Methods

### Maternal Vitamin D Deficiency Mouse Model

Five week old C57Bl/6J (Strain #000664) female mice (F0) purchased from the Jackson Laboratory were fed vitamin D deficient (VDD) or sufficient (VDS) diets for five weeks before mating to the control diet-fed male mice. The assigned diets were maintained throughout the subsequent pregnancy. VDD (0.0 IU/g vitamin D) and nutrient-matched VDS (1.0 IU/g vitamin D, D12450J) diets were obtained from Research Diets Inc. (10 kcal%fat, 20 kcal%Protein, and 70 kcal%Carbonate). VDD-fed females were supplemented with 1.5% calcium gluconate to maintain calcium homeostasis (20). Vitamin D deficiency of the F0 animals was confirmed by measuring serum 25-OH vitamin D levels using the Mouse Rat 25-OG Vitamin D ELISA kit (Eagle BIOSCIENCES) before mating and postnatal day 1 offspring (**Supplementary Figure 1A**). After delivery, all F0 mice were fed VDS diets. After weaning, the offspring (F1) were fed a VDS diet that was maintained until animal sacrifice. All animal studies were approved by the Institutional Animal Care and Use Committee at the Albert Einstein College of Medicine.

### Histopathological Study

After dissection, the liver samples were fixed in 4% paraformaldehyde solution at 4°C for overnight and processed for paraffin embedding. The paraffin-embedded tissue slides were stained with hematoxylin and eosin (HE), trichrome, and Gomori Reticulin stainings. We assessed 10 VDS-F1 and 19 VDD-F1 male mice.

### Transcription Analysis

We used male offspring for gene expression studies (n=6 VDD-F1 and n=6 VDS-F1). Total RNA was extracted from mouse livers at 16 weeks of age using the AllPrep DNA/RNA Micro Kits (QIAGEN). The RNA-seq libraries were generated using KAPA RNA HyperPrep with RiboErase kit (Roche) and sequenced on an Illumina NovaSeq sequencer (Novogene Co., Ltd., USA). After removing reads that failed the quality check and trimming adapter sequences, the resulting sequences were aligned to the mouse mm10 reference genome, quantifying gene expression by transcript counting (GENECODE release M15, GRCm38) using the STAR aligner (21). Differential expression as a result of dietary manipulation was determined using the Bioconductor package DESeq2 (22). We eliminated genes with fewer than the mean read counts per sample per gene is less than 12 from the analysis. Significantly differentially expressed genes (DEGs) were determined based on a log_2_-fold change of less than -1 or greater than 1 and a false discovery rate (FDR) adjusted p-value less than 0.05. DEGs were further assessed for their biological properties by Gene Ontology (GO) enrichment analysis using a Bioconductor package, clusterProfiler (23, 24). Quantitative reverse transcription PCR (qRT-PCR) was performed on the same RNA samples to verify the results. We used SuperScript III with random hexamer priming to synthesize cDNA and LightCycler FastStart Universal SYBR Green Master Mix for quantitative PCR. The measurement was performed using the LightCycler 480 system. The primers used in this study are listed in **Supplementary Table 1**.

### Cell Subtype Deconvolution

We used CIBERSORTx, a web interface-based tool, for quantifying the proportion of cell types in the RNA-seq data (25). A publicly available mouse liver (parenchymal and non-parenchymal liver cells isolated by a two-step collagenase perfusion) single-cell RNA-seq data (GSE134134) (26, 27) was used to generate a custom signature from single-cell liver gene expression data that represents the gene expression signatures of liver cell and immune cell subtypes. We combined four datasets of single-cell RNA seq libraries listed under GSE134134, 1000 (GSM3937757), 2000 (GSM3937758), 5000 (GSM3937759), and 10000 (GSM3937760) (26, 27). We used the Seurat R package for the single-cell RNA-seq analysis (28). We excluded cells with the detected number of genes were less than 1000 and reads from mitochondrial were greater than 10% before the analysis. We performed normalization and variance stabilization using the sctransform algorism (29), and the sctransformed datasets were merged using integration functions and identified clusters based on the gene expression profiles (29). We identified cell subtype clusters based on the expression status of maker genes (**Supplementary Figure 4**).

### Statistical Analysis

All statistical analyses were performed using R version 4.1.1 (https://www.r-project.org/). The Student’s *t*-test was used to analyze continuous variables between VDS and VDD unless otherwise stated, and Fisher’s exact test to analyze categorical variables. P-values of <0.05 or FDR-adjusted p-values in multiple testing analyses were considered significant.

## Results

### Prenatal Vitamin D Deficiency Is Associated With Histopathological Changes of the Livers of Offspring

**Figure 1:**
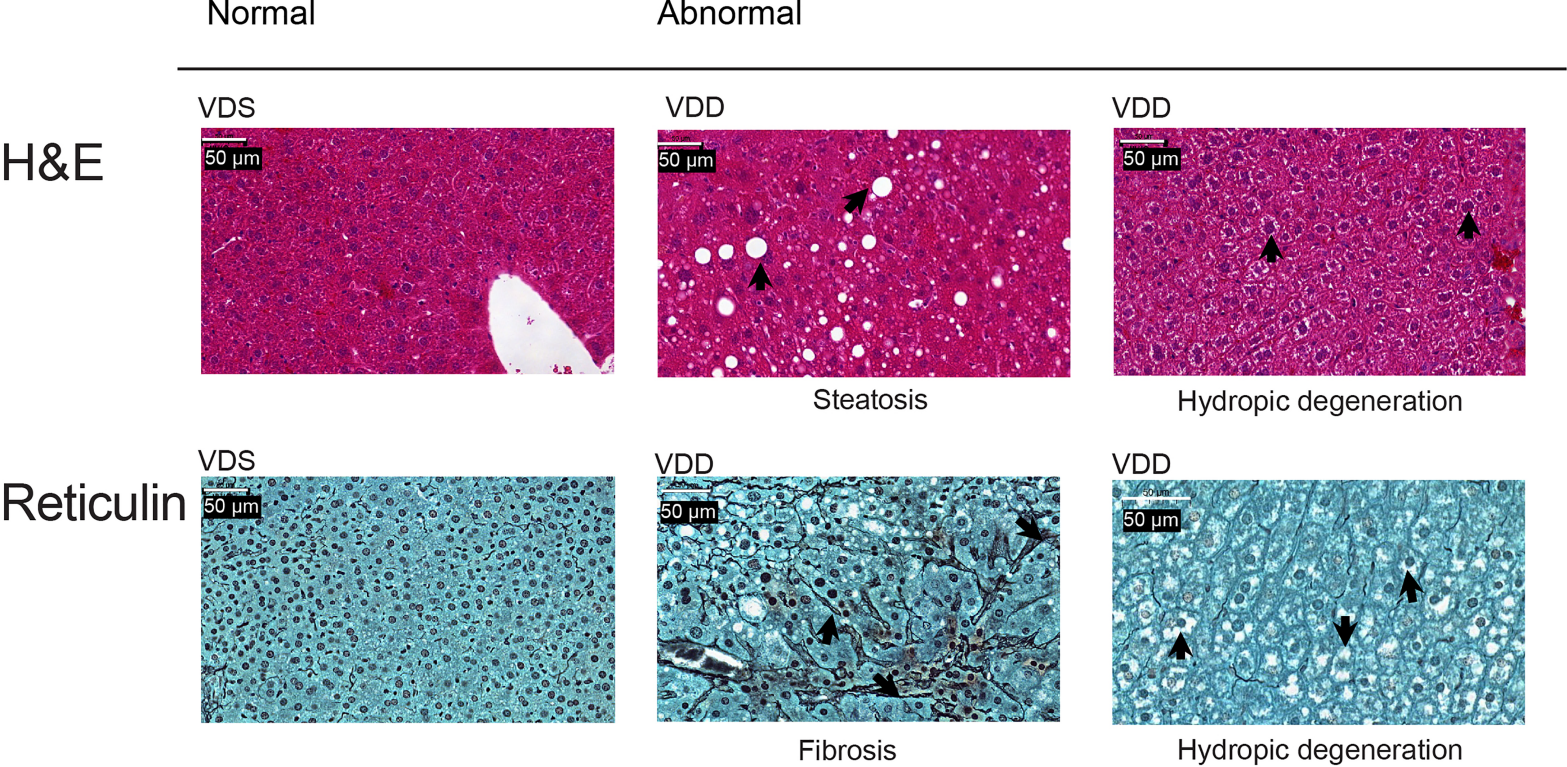
Representative histopathological alterations in the liver of VDD-F1 offspring. Top, hematoxylin-eosin stain; bottom, Gomori’s reticulin stain. The arrows indicate the described changes.

The growth trajectory of offspring was comparable in VDD-F1 and VDS-F1 (**Supplementary Figure 1B**). We dissected F1 male mice at 16 weeks of age to assess the long-term effects of prenatal vitamin D deficiency. We found histopathological alterations of the livers including hydropic degenerations, steatosis, and fibrosis in VDD-F1 offspring (**Figure 1**). While the prevalence rates were not statistically significant due to the small sample numbers, steatosis and fibrosis were only observed in the VDD-F1 offspring. The sizes of lipid droplets were varied between the individuals, but the morphology of the nuclei remained unaltered. We summarize the histopathological alterations in **Table 1**.

**Table 1:** A summary of histopathological alterations in liver.

	VDD-F1 (n=19)	VDS-F1 (n=10)	p-values*
Hydropic degeneration	11	4	0.675
Steatosis	1	0	0.063
Steatosis and Fibrosis	5	0	–
No alterations observed	8	6	0.562

*Fisher’s exact test.

### Prenatal Vitamin D Deficiency Is Associated With Permanently Altered Gene Expression of the Liver

**Figure 2:**
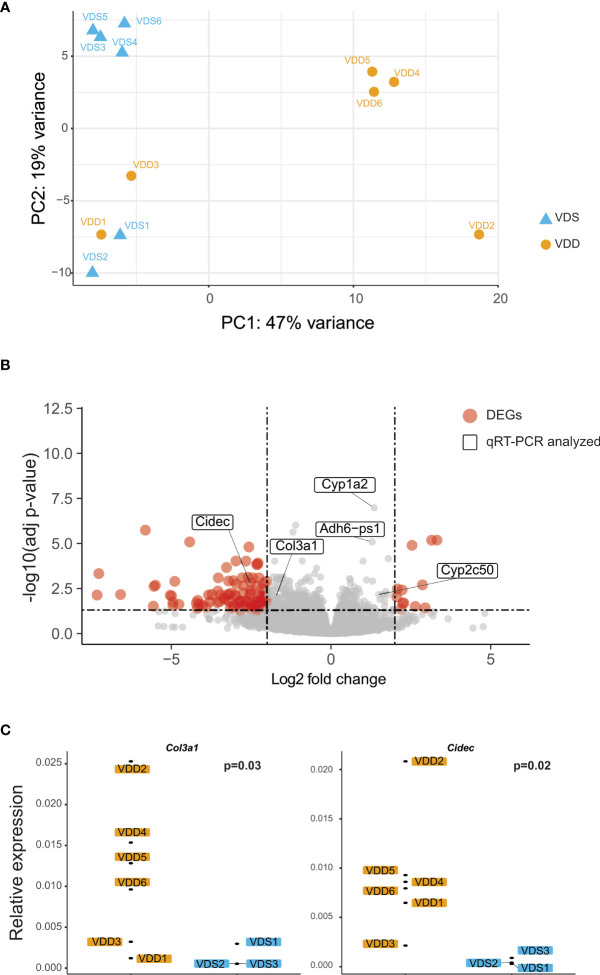
Prenatal vitamin D deficiency leads to altered gene expression in adult mouse liver. **(A)** PCA demonstrates variation in expression patterns between VDD and VDS offspring. **(B)** A volcano plot shows differentially expressed genes (DEGs) found in the analysis comparing mouse offspring exposed to a prenatal vitamin D deficiency versus mice exposed to a vitamin D sufficient diet. Genes are significantly differentially expressed if they have a p adjusted value less than 0.05 and -2 < fold change < 2. **(C)** qRT-PCR using cDNA generated from offspring liver samples validates the upregulation of the collagen, type III, alpha I (*Col3a1*), and *Cidec* genes.

The histopathological analyses suggested that prenatal vitamin D deficiency alters liver physiology. We, therefore, conducted a transcriptional analysis using bulk RNA-seq to assess the gene expression alterations linked to prenatal vitamin D deficiency. We extracted total RNA from the left lateral lobes of the liver and prepared libraries (n=6 per group, **Supplementary Figure 2**). We sequenced libraries at the depth of at least 26 million paired reads per library. After the quality check of the sequences and removal of low-quality reads, we performed a principal components analysis to compare the expression pattern between the individual F1 mice (**Figure 2A**, **Supplementary Table 2**). We observed three large clusters that consist of only VDS samples, VDD samples, and both VDS and VDD. There were no significant histopathological differences between VDD samples in different clusters. Next, we performed a differential expression analysis and identified 281 significant differentially expressed genes (DEGs) when comparing samples from mice who were exposed to prenatal VDD versus those exposed to prenatal VDS. 249 of DEGs were upregulated and 32 of DEGs were downregulated (**Figure 2B**, **Supplementary Table 3**). We verified the results using the same RNA samples and confirmed significant alterations or the same trends of DEGs by quantitative RT-PCR (**Figure 2C**, **Supplementary Figure 3**).

### The DEGs Are Enriched in Pathways for the Extracellular Structural Organization and Lipid Catabolism

**Figure 3:**
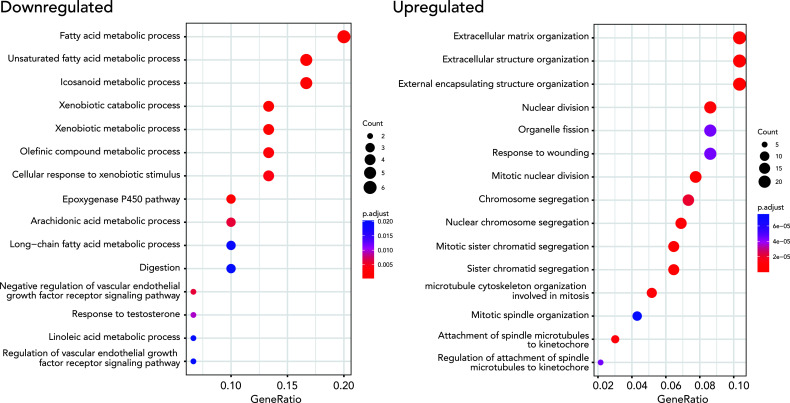
Results from GO enrichment analysis (biological processes) using a Bioconductor package, *clusterProfiler*, for up and down-regulated DEGs. The top 15 up (right) and downregulated (left) terms were. The size of the dots represents the number of the genes, and the color indicates the significance of the enrichment.

We performed gene ontology (GO) enrichment analyses on upregulated and downregulated DEGs respectively to test if the DEGs are enriched in specific gene pathways. We found that lipid metabolism-related pathways such as lipid catabolic process, xenobiotic metabolism, and linoleic acid metabolic process are enriched in down-regulated DEGs. 249 upregulated DEGs were enriched in extracellular structure organization, nuclear division, and mitosis-related pathways (**Figure 3**, **Supplementary Table 4**).

### Cell Subtype Proportion Analysis of Bulk RNA-Seq Data Revealed That Prenatal Vitamin D Deficiency Alters Cell Subtype Composition of Non-Parenchymal Liver Cells

**Figure 4:**
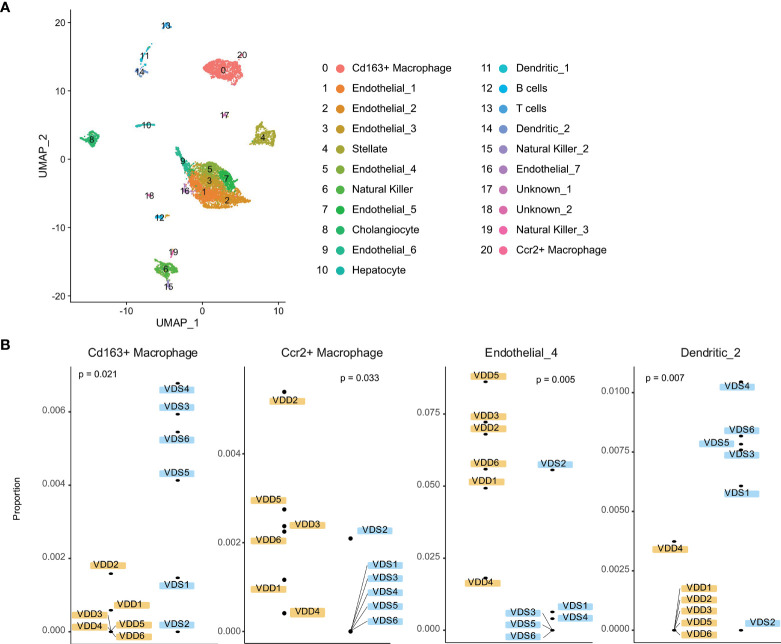
Liver cell subtypes were found in different cell subtype proportions in prenatal VDD compared to VDS offspring. **(A)** Publicly available single-cell RNA-seq data was processed and clustered as shown in the UMAP plot. Gene expression patterns of clusters were used to define cell identities. **(B)** The proportions of Cd163+ macrophages and Ccr2+macrophages were inversely related between prenatal VDD and VDS offspring. Dendritic cells were found in a significantly lower proportion in VDD samples while a subset of endothelial cells was found in a higher proportion in VDD samples.

We next tested if the proportions of cell subtypes are affected based on prenatal exposure to VDS or VDD. To perform cell subtype proportion analysis, we generated reference expression signatures of each liver cell subtype using publicly available adult liver single-cell RNA-seq data. We segregated 21 clusters of the adult liver cells, as shown in **Figure 4A** and **Supplementary Figure 4**. Based on the relative expression of well-known marker genes of liver cell subtypes identified by the mouse cell atlas, we identified the cell subtypes of each cluster. The cluster information was then used to generate a custom signature of gene expression in liver cells using CIBERSORTx. The generated reference expression signature was used to perform deconvolution of bulk RNA-seq data of each sample (**Supplementary Table 5**). As we expected, the hepatocyte proportion was the highest among the cell subtypes (80.6% VDD and 81.4% VDS, p=0.39) (**Supplementary Table 6**). While the proportions of hepatocytes were not significantly altered, we observed significant alterations in non-parenchymal liver cell subtypes, macrophages, endothelial cells, and dendritic cells (**Figure 4B**). Interestingly, while Cd163-positive macrophages were reduced in prenatal VDD offspring liver, Ccr2-positive macrophages were increased compared to that of VDS offspring. We previously reported that cell subtype composition significantly contributes to the gene expression profile (30). We therefore tested if the DEGs we identified were attributable to the cell subtype proportion differences between samples. We did not observe significant contributions of cell subtype proportion variations to the gene expression profile (**Supplementary Figure 5**). This result indicates that the identified DEGs were independent of the cell subtype proportion variations.

## Discussion

In this study, we found that prenatal vitamin D deficiency alters liver histology and transcription profiles in offspring adulthood. We observed livers with varying ranges of cytoplasmic clearing, hydropic changes, and fibrosis in histopathological analysis. Transcriptional analysis showed that lipid metabolism-related genes, mitosis-related genes, and extracellular structure organization-related genes were enriched in DEGs. Moreover, cell subtype deconvolution analysis indicates alterations of non-parenchymal liver cell proportions. These findings suggest that prenatal vitamin D deficiency permanently alters the physiological and metabolic properties of offspring livers.

Herrick et al. reported that more than 23% of Americans equal or older than 1 year are at risk of vitamin D inadequacy or deficiency and the prevalence of the risk of deficiency is at its highest among adults among 20-39 years old using 2011-2014 National Health and Nutrition Examination Survey (NHANES) data sets (1). They also reported that the risk of deficiency was higher among non-Hispanic Black than non-Hispanic Asian, non-Hispanic White, and Hispanic participants. Moreover, the high prevalence of vitamin D deficiency and inadequacy is associated with obesity (5, 31, 32). These findings suggest that reproductive-age women with obesity have a higher risk of vitamin D deficiency during pregnancies, specifically women of the vitamin D deficiency vulnerable ethnic/race groups.

The associations between maternal vitamin D deficiency and adverse maternal and fetal outcomes have been well reported (33–37). A systematic review of 3357 studies revealed that maternal vitamin D deficiency increases the risks of gestational diabetes, preeclampsia, and small for gestational age infants (35). In addition, prior studies have implicated associations between maternal vitamin D deficiency and offspring obesity later in life in both human observation studies (14–17, 38) and dietary manipulation studies in rodents (18, 39). We did not observe an obese phenotype; however, we found that mice exposed to prenatal vitamin D deficiency showed transcriptional alterations in lipid metabolism-related pathways. Moreover, the histopathological analysis suggested that exposure to a prenatal vitamin D deficiency affects the physiology of the liver at the adult stage. Our results suggest the existence of long-term memory of prenatal vitamin D deficiency exposure in the liver.

The association between vitamin D deficiency in adulthood and chronic liver diseases (CLD) (40), including cirrhosis (41), non-alcoholic fatty liver disease (NAFLD, recently been proposed to rename as metabolic-associated fatty liver disease [MAFLD) (42)] (43–45), and non-alcoholic steatohepatitis (NASH) (44, 45), have been identified in many cross-sectional studies. Stokes et al. reviewed the associations between CLD and vitamin D deficiency (46). We fed a vitamin D sufficient diet to all offspring after weaning, finding that the serum vitamin D concentration of mice exposed to prenatal VDD became normal by 5 weeks of age. Therefore, the pathogenesis of the liver phenotype we observed in the liver of prenatal VDD offspring was not by deficiency at the time, likely by a long-term memory of VDD exposure. However, there are several similarities that exist. For instance, the infiltration and activation of macrophages is a key feature of endoplasmic reticulum stress-induced inflammation in liver injury, with VDR signaling regulating the inflammatory response in macrophages (47). We found that two distinct macrophage proportions, Cd163-positive, the anti-inflammatory (M2 phenotype) macrophages (48), and Ccr2-positive macrophages had distinct patterns of alteration in prenatal VDD offspring. Associations between the Ccr2-positive Kupffer cells (hepatic residential macrophage) and choline-deficient amino acid-defined diet-induced hepatic steatosis and fibrosis have been identified (49, 50). Several studies reported that a high-fat diet-induced hepatic steatosis increases bone-marrow (BM)-derived macrophages, which predominantly express Ccr2 (51, 52). The cell subtype reference expression signatures of both Cd163-positive and Ccr2-positive macrophages in this study showed higher expression of *Emr1* (F4/80) but low expression of *Itgam* (Cd11b) (52), indicating that they may be tissue-resident macrophages. Ccr2 expression in Kupffer cells contributes to BM-derived macrophage recruitment (49). The resident macrophages are derived from multiple anatomical locations during development suggesting developmental origin cell subtype proportion alterations may exist in the liver that is associated with a predisposing condition of prenatal VDD to the liver phenotype at the adult stage. The GO enrichment analyses on DEGs in this study showed enrichment of biological processes GO terms including extracellular matrix organization, regulation of vascularized development, and regulation of angiogenesis, processes which occur with liver fibrosis. Our study provides new insight into how prenatal VDD affects the liver phenotype and physiology as well as how this results in changes in the proportions of liver-resident immune cell subtypes in adult offspring.

There are several limitations of the current study that we would like to address in the future. First, we performed dietary manipulation on F0 female mice for five weeks prior to mating until the delivery. Belenchia et al. reported that it takes at least two weeks for serum vitamin D levels to return to normal after switching the diet to VDS from long-term VDD feeding (53). Thus, the F1 offspring were exposed to vitamin D deficient and insufficient conditions for the first two weeks of life. Using foster mothers would help us dissociate the effects between prenatal and neonatal periods. Second, while we observed cell subtype proportion changes in the prenatal VDD offspring, we cannot conclude if this alteration contributes to prenatal VDD exposure or results from liver damage. We believe testing cell subtype proportions in the liver before liver injury started would resolve this question.

Moreover, we estimated cell subtype proportion changes by the bulk RNA-seq results; therefore, the estimated proportion may be affected by the expression changes in specific cell subtypes. Testing the expression profiles at the single-cell levels will test if the alterations we observed in this study are attributed to the gene expression alterations between the same cell subtypes. Lastly, Wang et al. estimated the relationship between mice and humans at stages of growth and reported that the age range of 10 to 64.29 weeks of age in mice corresponds to the adult stage in humans (20 to 51 years old) (54), suggesting 16 weeks of age is at the early adult stage. The prevalence of NAFLD increases after age 30 (55) and age older than 40 is a risk factor of NAFLD (56). This study could be improved by maintaining the mice for longer than 16 weeks of age following prenatal VDD in order to observe more changes related to prenatal VDD and at the adulthood stage.

In summary, we found that prenatal vitamin D deficiency alters gene expression profiles of the liver and cell subtype proportions of non-parenchymal cells at the adult stage. The prevalence of at risk of vitamin D deficiency and insufficiency is high among reproductive-age women, and a significant association of obesity to low vitamin D status has been implicated. Childhood obesity in the world is rising in frequency, with the prevalence of overweight/obesity among children aged 5-18 in the world rising from 4% in 1974 to 18% in 2016 (57). Epidemiological studies have identified significant associations between excess weight during childhood and the risk of adulthood obesity (58–62). Moreover, the incidence of NASH and NAFLD/MAFLD in young people has increased to 1.52 times between 1990 and 2017, and the prevalence of NAFLD/MAFLD in children varies by ethnicity (56). Prenatal and neonatal vitamin D deficiency may be contributing to this increase in prevalence rates. Reducing maternal vitamin D deficiency could reduce the adverse health outcome of the next generation, while understanding mechanisms of how our body remembers prenatal vitamin D status over the lifespan should help us to find interventions and treatments to prevent developmental origins of health and diseases in the liver.

## Data Availability Statement

The original contributions presented in the study are publicly available. This data can be found here: GEO, GSE194417.

## Ethics Statement

The animal study was reviewed and approved by Institutional Animal Care and Use Committee at the Albert Einstein College of Medicine.

## Author Contributions

Conceptualization, MS. Methodology, KL, RD-T, and MS. Formal analysis, KL and GE-B. Histopathological analysis support, JO. Histopathological analysis, JFG. Data curation, KL and MS. Writing—original draft preparation, KL and MS. Writing—review and editing, KL, JFG, RD-T, JMG, and MS. Visualization, KL, GE-B, and MS. Supervision, JFG, JMG, and MS. Funding acquisition, MS. All authors have read and agreed to the published version of the manuscript.

## Funding

This work was supported by the Human Genome Project Pilot Grant of the Department of Genetics at Albert Einstein College of Medicine and partially supported by the National Institutes of Health under award number R01HL145302 (MS).

## Author Disclaimer

The content is solely the responsibility of the authors and does not necessarily represent the official views of the National Institutes of Health.

## Conflict of Interest

The authors declare that the research was conducted in the absence of any commercial or financial relationships that could be construed as a potential conflict of interest.

## Publisher’s Note

All claims expressed in this article are solely those of the authors and do not necessarily represent those of their affiliated organizations, or those of the publisher, the editors and the reviewers. Any product that may be evaluated in this article, or claim that may be made by its manufacturer, is not guaranteed or endorsed by the publisher.
