# Supplementary material for: Vitamin D Deficiency During Development Permanently Alters Liver Cell Composition and Function
Source: Front Endocrinol (Lausanne). 2022 May 12;13:860286. doi: 10.3389/fendo.2022.860286 (PMC9133936; doi:10.3389/fendo.2022.860286)
Supplement: Supplementary file 1 [file DataSheet_1.pdf]

Supplementary Figure 1

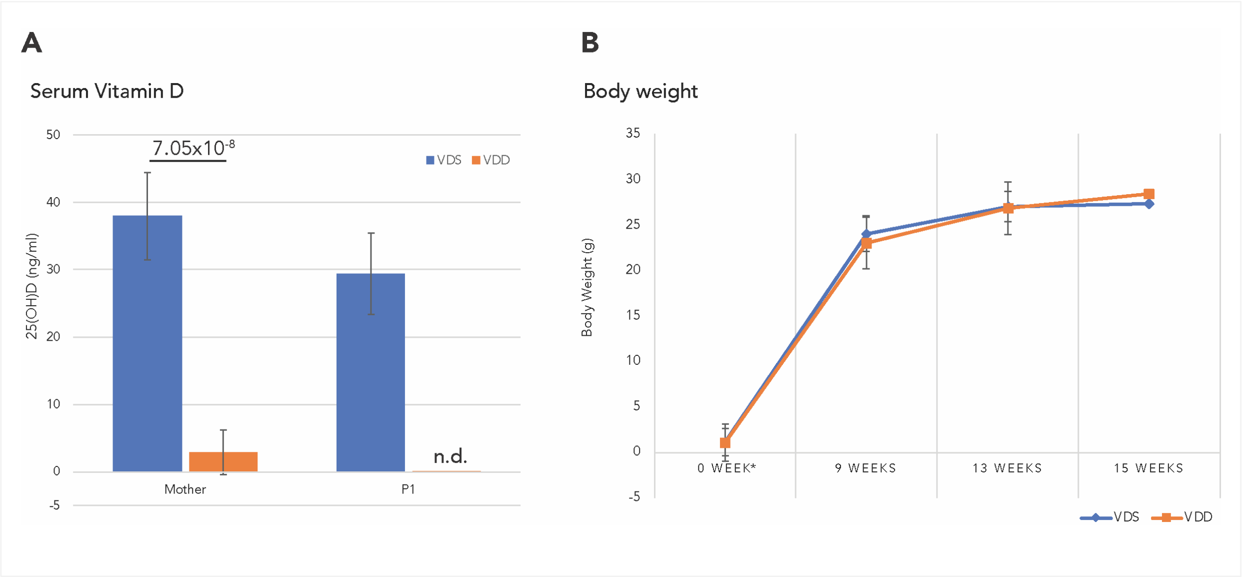

Supplementary Figure 1: Serum vitamin D concentrations of mother and postnatal day 1 offspring (A), and body weight trajectory of offspring (B)

A: The serum vitamin D (25-OH vitamin D) levels were measured using the Mouse Rat 25-OG Vitamin D ELISA kit (Eagle BIOSCIENCES) at 6 weeks after feeding vitamin D deficient (VDD)- or sufficient (VDS)-diets (before the mating) and postnatal day1 offspring. The serum vitamin D levels of postnatal day 1 offspring born to VDD fed mother was out of the detection limit (4.78 ng/ml). B: We measured the bodyweight of offspring at 0 (postnatal day 1), 9, 13, and 15 weeks of age. We did not observe any significant alterations between VDD and VDS at any time point.

Supplementary Figure 2

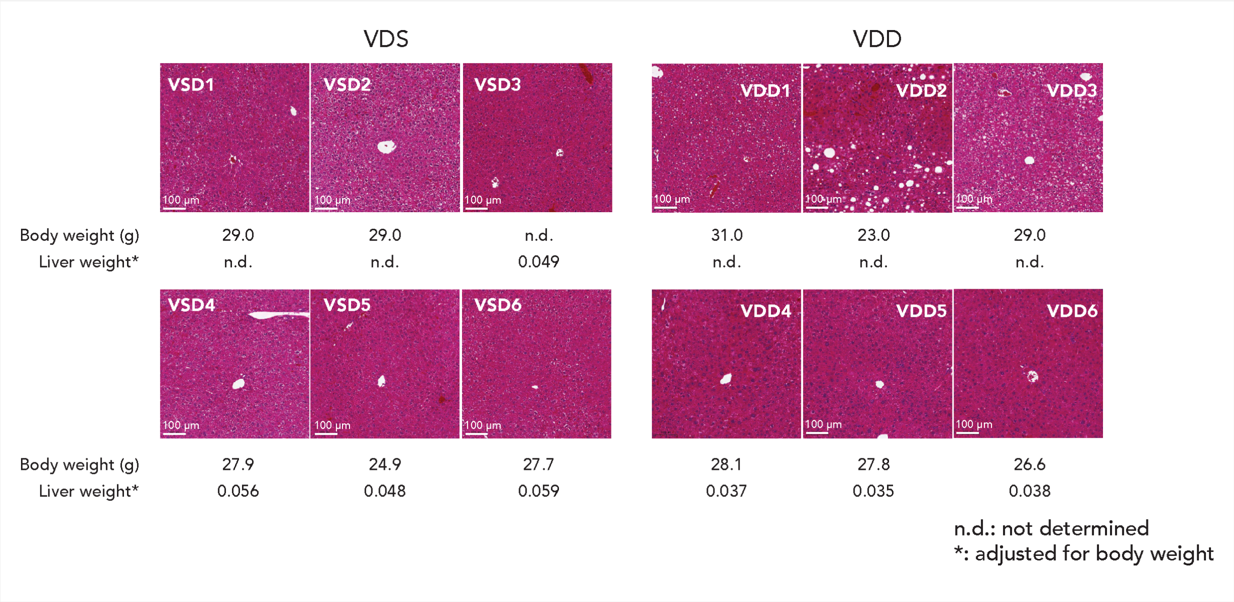

Supplementary Figure 2: Histopathological representations of F1 offspring with body weight (g) and body weight adjusted liver weight

We showed H&E staining slides of each sample we used in our RNA-seq analysis. The body weight was obtained at the sampling time (16 weeks of age), and the liver weight was adjusted for the bodyweight of the mice.

Supplementary Figure 3

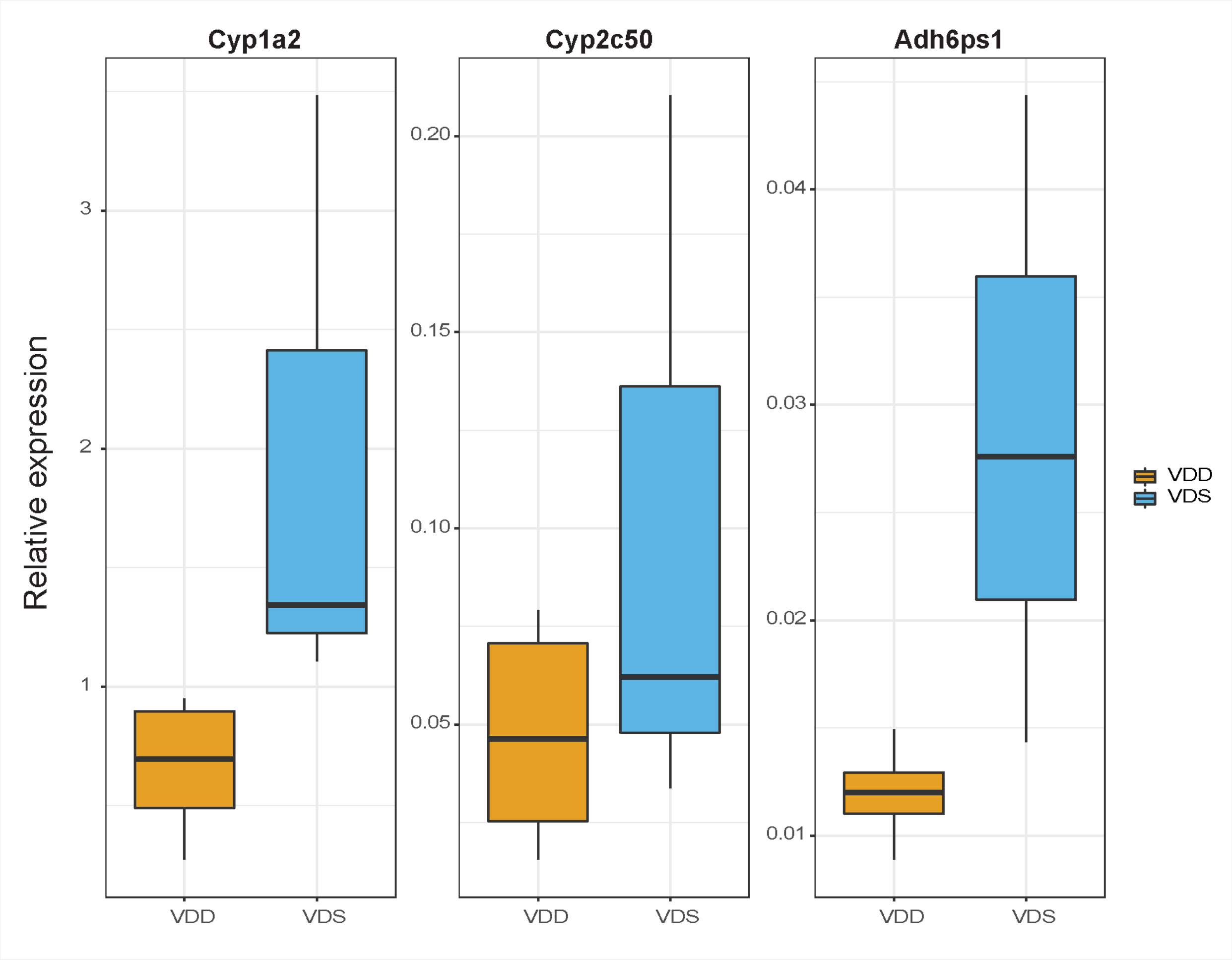

Supplementary Figure 3: Results of quantitative RT-PCR of differentially expressed genes

We performed quantitative RT-PCR analyses on the same RNA samples we used for the RNA-seq library preparation. Relative expression was calculated as GAPDH normalized expression status. The box indicates the first and third quartile, the bar in the box indicates the median, the tips of the vertical line indicate the minimum and maximum values. Due to the sample availability, we only have 3 VDS and 6 VDD samples (Cyp1a2: p=0.22, Cyp2c50: p=0.42, and Adh6ps1: p=0.92).

Supplementary Figure 4

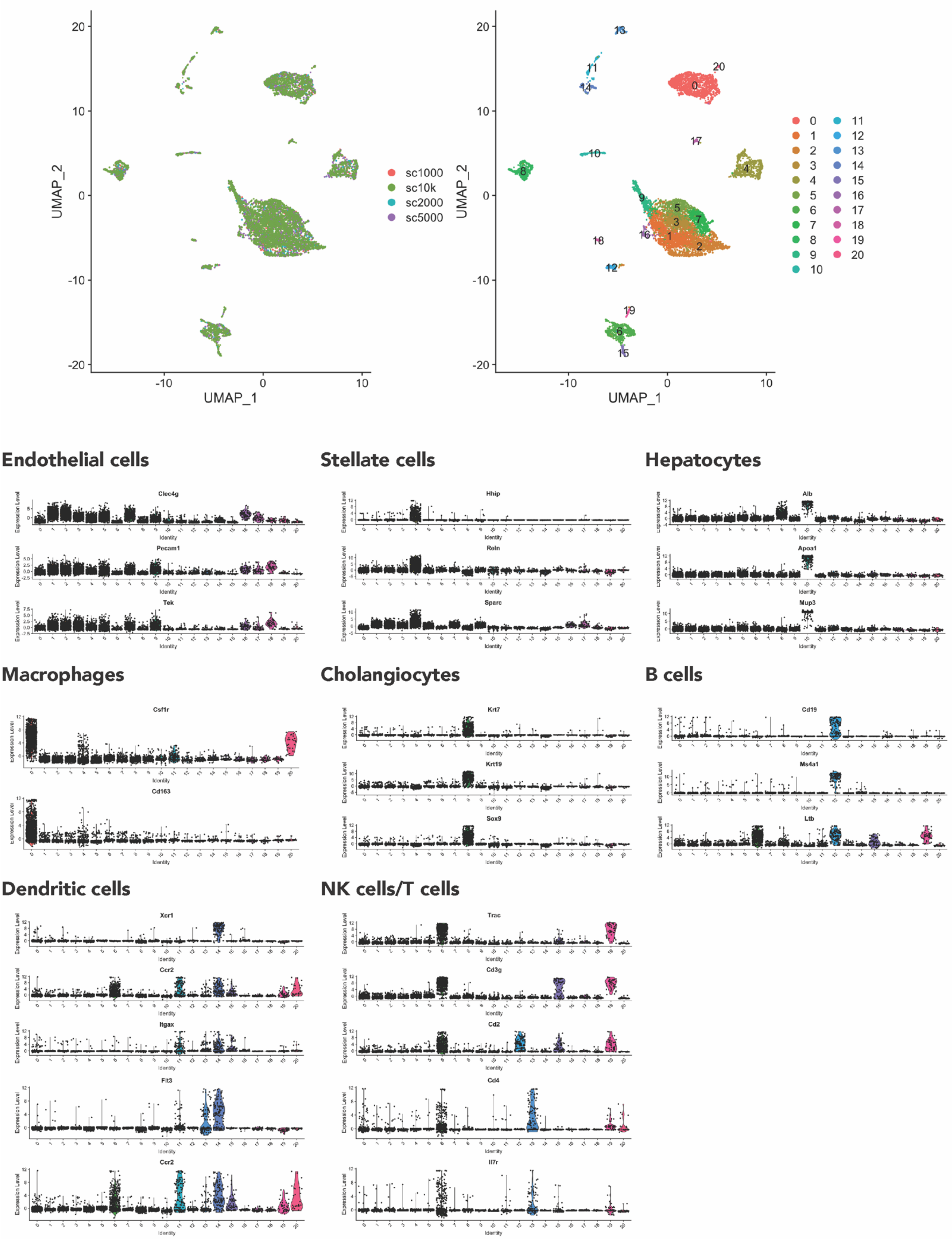

Supplementary Figure 4: Identification of cell subtypes of single-cell RNA-seq expression signature profile

Top: UMAP plots by dataset (left) and by identified clusters based on gene expression profile (right); Bottom: Marker gene expression status of each cell cluster

Supplementary Figure 5

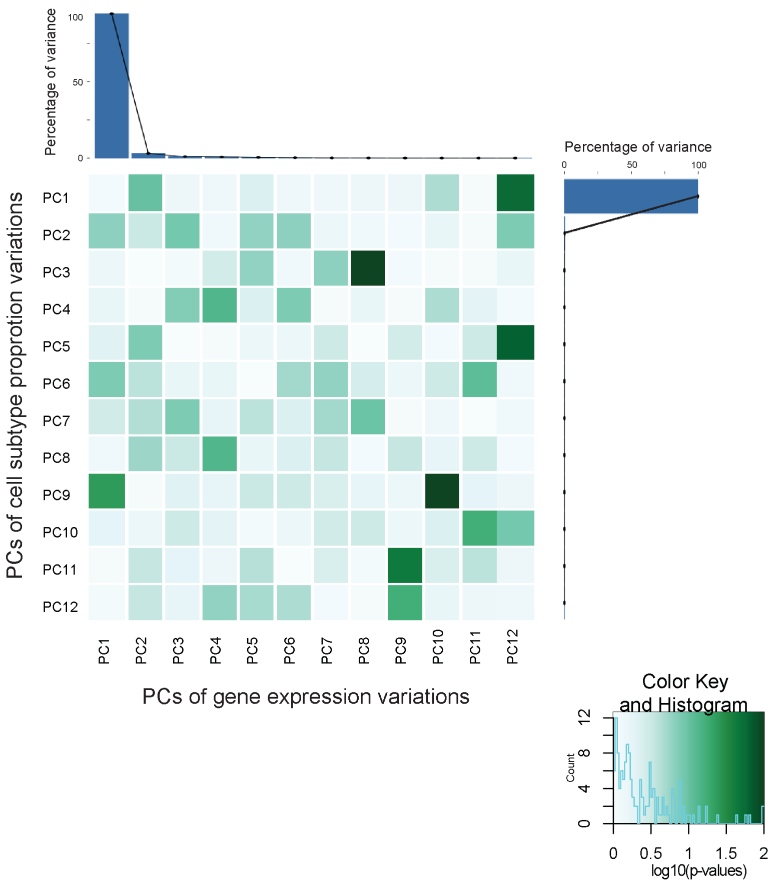

Supplementary Figure 5: The associations between gene expression variations and cell subtype proportion variations

We calculated the significance of contributions of each PC of gene expressions and PC of cell subtype proportion variations. Both PC1 of gene expression and PC1 of cell subtype proportions accounted for more than 99% of the variance of each variation. We did not observe a significant association between them.
